# A Methodology to Monitor Airborne PM_10_ Dust Particles Using a Small Unmanned Aerial Vehicle

**DOI:** 10.3390/s17020343

**Published:** 2017-02-14

**Authors:** Miguel Alvarado, Felipe Gonzalez, Peter Erskine, David Cliff, Darlene Heuff

**Affiliations:** 1 Environment Centre, Sustainable Mineral Institute, The University of Queensland, 4072 Brisbane, Australia; p.erskine@uq.edu.au; 2 Science and Engineering Faculty, Queensland University of Technology (QUT), 4000 Brisbane, Australia; felipe.gonzalez@qut.edu.au; 3 People Centre, Sustainable Mineral Institute, The University of Queensland, 4072 Brisbane, Australia; d.cliff@mishc.uq.edu.au; 4 Advanced Environmental Dynamics Pty Ltd., Ferny Hills, 4055 Queensland, Australia; darlene.heuff@aedconsultants.com.au

**Keywords:** PM_10_, monitoring, blasting, unmanned aerial vehicle (UAV), multi-rotor UAV, optical sensor

## Abstract

Throughout the process of coal extraction from surface mines, gases and particles are emitted in the form of fugitive emissions by activities such as hauling, blasting and transportation. As these emissions are diffuse in nature, estimations based upon emission factors and dispersion/advection equations need to be measured directly from the atmosphere. This paper expands upon previous research undertaken to develop a relative methodology to monitor PM_10_ dust particles produced by mining activities making use of small unmanned aerial vehicles (UAVs). A module sensor using a laser particle counter (OPC-N2 from Alphasense, Great Notley, Essex, UK) was tested. An aerodynamic flow experiment was undertaken to determine the position and length of a sampling probe of the sensing module. Flight tests were conducted in order to demonstrate that the sensor provided data which could be used to calculate the emission rate of a source. Emission rates are a critical variable for further predictive dispersion estimates. First, data collected by the airborne module was verified using a 5.0 m tower in which a TSI DRX 8533 (reference dust monitoring device, TSI, Shoreview, MN, USA) and a duplicate of the module sensor were installed. Second, concentration values collected by the monitoring module attached to the UAV (airborne module) obtaining a percentage error of 1.1%. Finally, emission rates from the source were calculated, with airborne data, obtaining errors as low as 1.2%. These errors are low and indicate that the readings collected with the airborne module are comparable to the TSI DRX and could be used to obtain specific emission factors from fugitive emissions for industrial activities.

## 1. Introduction

The use of Unmanned Aerial Vehicles (UAVs) to collect environmental data has increased exponentially in only a few years. Their capacity to reach places where humans could be at risk or it could become too expensive for small operations, makes them very useful for gas and particulate monitoring in the mining industry. Several authors have published articles where progress on the use of UAVs for industrial use or investigation has been made by using two UAVs simultaneously [1], combining multiple gas sensors and imagery [2,3], using gas tracking algorithms [4,5,6], using solar energy [7], and making use of microwave sensors [8].

The methodology applied and tests undertaken for this investigation are a continuation of the work published by Alvarado et al. [9,10]. Improvements to the methodology include: the development of a new modular sensor, focusing only on the use of a multi-rotor UAV with a tailored sampling probe, and the use of a 5 m tower to validate the PM_10_ readings obtained by the UAV. The emission rates obtained from a point source created for the flight tests, were evaluated to determine the capability of the methodology to be used at open pit mine sites to estimate emission factors. In the following sections these experiments are described and their results discussed.

## 2. Experimental Development

### 2.1. Dust Monitoring Module

Following Alvarado et al. [9,10] a sampling module and data logger transmission system were developed using the OPC-N2 particle counter from Alphasense (Great Notley, Essex, UK) [11] with a minimum response time of 0.2 s. In addition to the particle counter the dust monitoring module is integrated with a:

- Barometer, temperature and humidity sensor, BME280 (Bosch, Reutlingen, Germany);
- Raspberry Pi 3 board (Raspberry Pi Foundation, Cambridge, UK);
- uBlox LEA6 GPS (uBlox, Thalwil, Switzerland);
- XBee 2.4 GHz Radio (XBee, Minnetonka, MN, USA);
- 3DR Airspeed Sensor (3DR, Berkeley, CA, USA);
- Lipo battery (3.7 V) (3DR, Berkeley, CA, USA).

**Figure 1:**
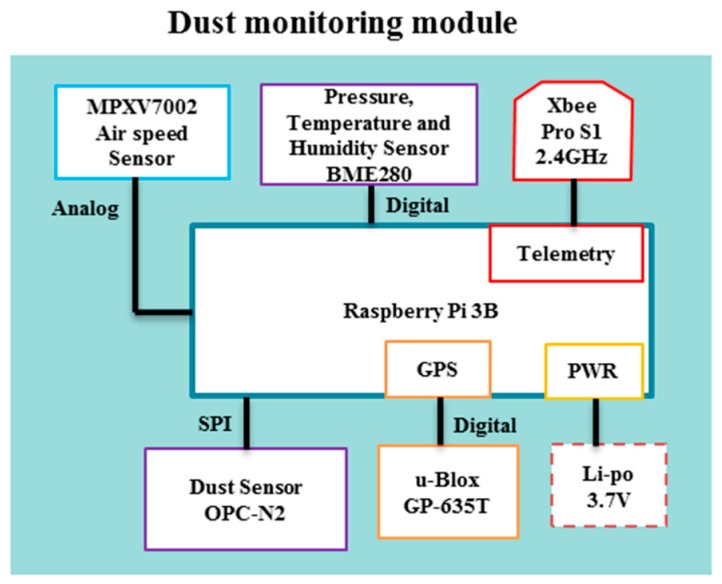
Architecture of dust monitoring module.

The architecture of the dust monitoring module is presented in Figure 1. Information from each sensor (e.g., dust, pressure, temperature and humidity) was sent to a ground station via the XBee 2.4 GHz radio for monitoring, and recorded in a micro-SD card used by the Raspberry Pi. The dust monitoring module was programmed to have a response time of 0.2 s. Time synchronisation was mainly achieved using the GPS time stamp of each reading. The name of each log file was also recorded with the time and date synchronised with the internet using a portable WiFi hot spot.

**Figure 2:**
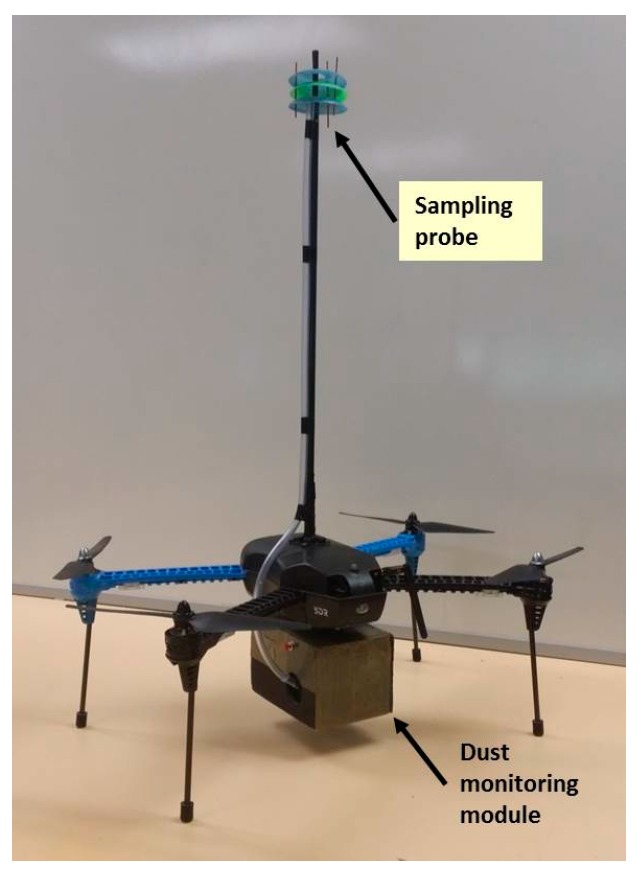
Physical configuration of the multi-rotor UAV with the sampling probe and dust monitoring module attached.

The total weight of the dust monitoring module was of 382.0 g and had the following dimensions: 14.0 cm × 9.0 cm × 10.0 cm. The small multi-rotor UAV used for the investigation was an IRIS+ from 3DR (Berkeley, CA, USA), with an endurance of approximately 10 min. The IRIS+ multi-rotor UAV was the selected flying platform due to robustness, user-friendly controls, versatility and ability to be modified and serviced. The IRIS+ was adapted to carry an air sampling probe and an enclosure for the dust monitoring module as shown in Figure 2.

### 2.2. Aerodynamics Downwash/Upwash Experiment and Analysis

When using a multi-rotor UAV two main issues are identified when obtaining a representative air sample from the atmosphere: difficulty of producing isokinetic flow, and designing an air sampling probe and determining the best position in the multi-rotor UAV.

To understand the disturbance produced by the propellers in the volume of air that will be sampled, an aerodynamic experiment was designed. Through this experiment, it was possible to observe the behavior of the volume of air surrounding the multi-rotor UAV and determine if isokinetic flow would be possible. Isokinetic flow is an important consideration when monitoring or sampling for particles below 10 µm in diameter, otherwise the resulting value could be under or over-predicted [12]. However, being able to obtain isokinetic flow in non-static conditions can be extremely difficult [13,14]. Air flow intake needs to be controlled so the air streamlines are unaltered during the transportation of particles from the atmosphere past the probe’s nozzle. To achieve these conditions several factors have to be considered: air velocity has to be equal inside and outside the sampling nozzle, air intake has to have the same direction (iso-axial), and has to be placed out of the air mixing zone [14,15,16]. Other variables to consider for isokinetic measurements with aircrafts are: diffusion, sedimentation, and turbulent inertial deposition. However, the variables considered will vary depending on the type of aircraft [15,16,17]. Related literature on the topic mainly focuses on fixed wing UAVs which, due to their flight characteristics, can provide better conditions for isokinetic sampling [16,17]. Factors such as angled flight, static flight or hovering, and constant change in wind direction, make achieving isokinetic conditions very challenging for multi-rotor UAVs. Von der Weiden et al. [12], used a different approach due to the difficulty of sampling in isokinetic conditions. They created a correction calculator for air sampling that does not meet the criteria necessary to be isokinetic and iso-axial. The calculator considers variation in sedimentation, deposition due to inlets, bends, contractions, and diffusion factors. However, their approach was only used and tested for the fixed wing UAV specified in their study.

**Figure 3:**
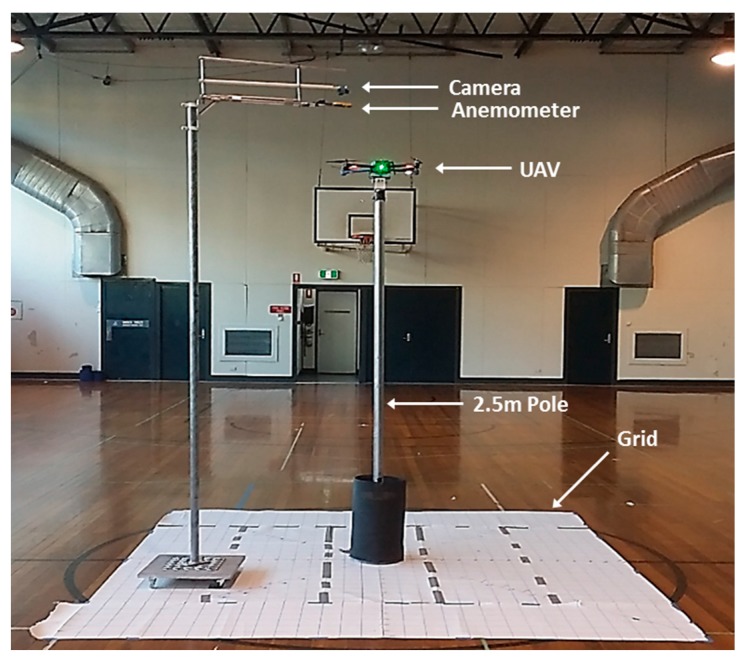
Set up of the multi-rotor UAV and airspeed measuring pole for aerodynamics test.

**Figure 4:**
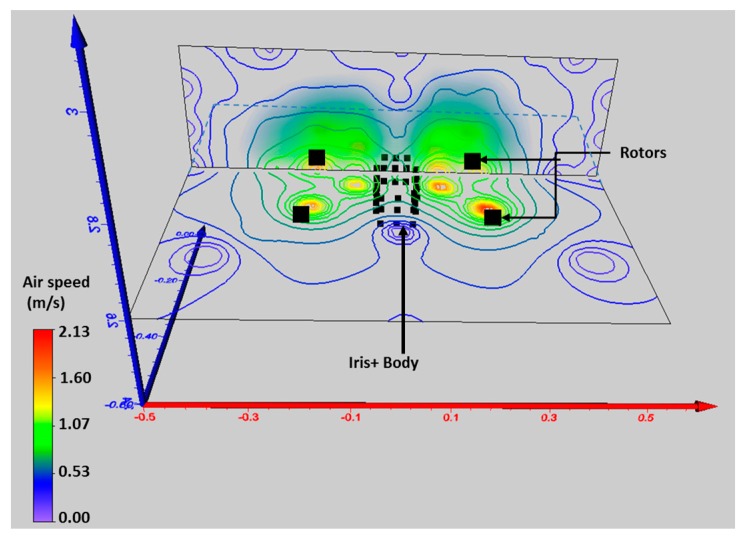
3D visualization of the different airspeed regions produced by the propellers upwash at the top and sides of the multi-rotor UAV. Axis units in meters.

For the aerodynamics experiment, the UAV was placed indoors and mounted on a 2.5 m pole. Measurements were taken with a Kestrel 2500 anemometer (Minneapolis, MN, USA) which was positioned around the UAV following a grid pattern at different x, y and z positions (Figure 3). With the readings collected, a 3D visualization map was generated to observe the airflow produced by the propeller downwash and upwash (Figure 4).

It was determined that the downwash produced by the multi-rotor UAV does not generate a uniform pattern by observing the readings and visualisations. The downwash varies by forming a cone which increases its base as distance from the center of the UAV increases (Figure 4). The upper side of the UAV on the other hand has a constant air speed flux which drops after a distance of approximately 40.0–45.0 cm. This constant air flux is also observed to the sides of the UAV up to a distance of 40.0–45.0 cm. The constant mixed air boundaries around the multi-rotor UAV helped define the position of the probe on the side of the UAV (horizontally) or on the top of it (vertically). A horizontal probe would increase the capacity of the UAV to collect an isokinetic sample when the wind direction is in line with the direction of the probe. However, when conducting filed tests, wind direction varied up to 68° during the testing period (10 min approximately) for three out of four flights conducted. Such ample variation in the intake angle is a considerable change of the ideal conditions required for isokinetic sampling. To overcome this issue, it was decided to use the vertical probe on top of the UAV at a distance of 47.5 cm from the center of the UAV (Figure 2). The use of a canopy on the top also increases its capacity to retain small volumes of the air targeted. This can be useful in high wind speeds. A vertical probe has the same exposure when taking air samples regardless of the orientation of the multi-rotor UAV or the wind flow.

### 2.3. Calcined Alumina Correction Factor

**Figure 5:**
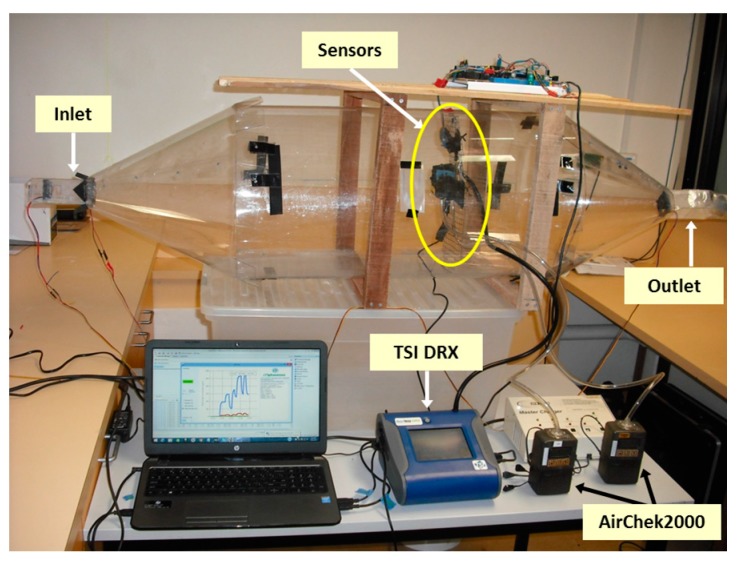
Set up of monitoring devices and dust chamber for calcined alumina calibration tests.

**Figure 6:**
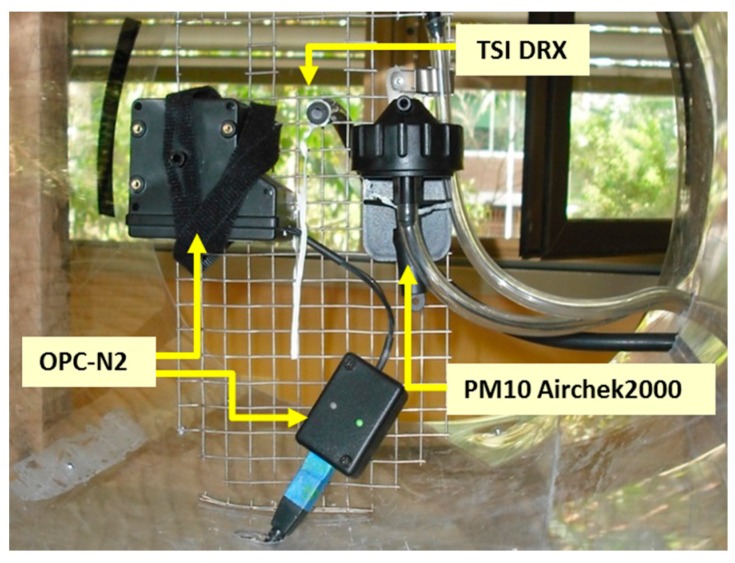
Layout of all monitoring equipment and sensors used to obtain the particle correction factor.

A correction factor was calculated for the dust source so the data collected by the sensing module could be used for further estimates. The source of dust chosen for the test was calcined alumina, which is less hygroscopic than talcum powder, used in previous tests [9]. Being less hygroscopic helped keep the optical sensors free of dust adhering to the components and accumulating inside the protective casing. In addition, according to the particle size distribution of the calcined alumina used for the experiment, the dust had a PM_10_ content of approximately 30%. This value is ten times higher than the average PM_10_ content found in talcum powder. A higher PM_10_ content allowed testing the sensor with small amounts of dust. The highest concentration of PM_10_ measured during the experiment was of 17.32 mg/m^3^ (19.42 mg/m^3^ of TSP). An OPC-N2 optical particle counter and a TSI DRX 8533 [18] were collocated with a gravimetric sampler, an AirChek2000 (Eighty Four, PA, USA), to calculate their correction factor (Figure 5 and Figure 6). Concentrations from the OPC-N2 and the TSI DRX were obtained directly from the logging files produced by the interface software provided by the manufacturers. Tests were done in a dust chamber of approximately 0.104 m^3^.

The tests consisted of inducing dust into the chamber with the aid of an axial fan. Three tests were conducted using a regular supply of dust at intervals lasting 3 min for the first, 6 min for the second and 9 min for the third test. In addition to the tests where dust was supplied, two blank tests were conducted to know background dust levels, resulting in an average concentration of 4.82 µg/m^3^ of PM_10_. Once all the data was collected, lineal regression analysis was used to calculate the correction factors between devices.

**Figure 7:**
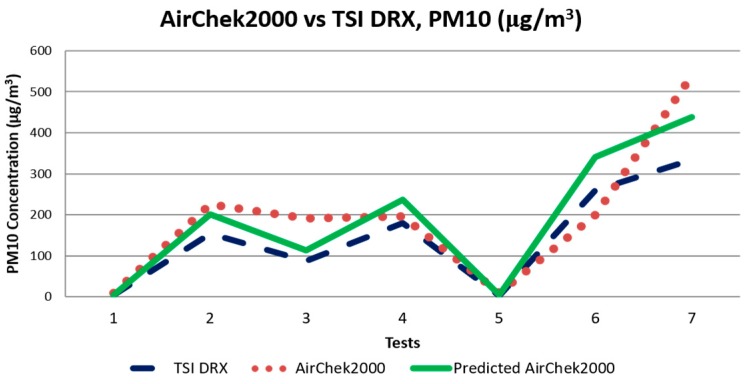
Correlation obtained to calculate the correction factor for the TSI DRX against the gravimetric sampler.

The samples collected with the AirChek2000 were used as the main reference. The filters used by the sampler were sent to a certified laboratory (ALS Environmental, New Castle, Australia) for analysis and determination of calcined alumina PM_10_ concentrations. The correction factors calculated are shown in Table 1. Figure 7 shows the resulting predicted values for the AirChek2000 making use of the particle correction factor calculated with the correlation between the TSI DRX and the air sampler. A coefficient of determination (R^2^) of 0.92 for the correlation indicates a good fit between the optical sensor and the air sampler.

**Figure 8:**
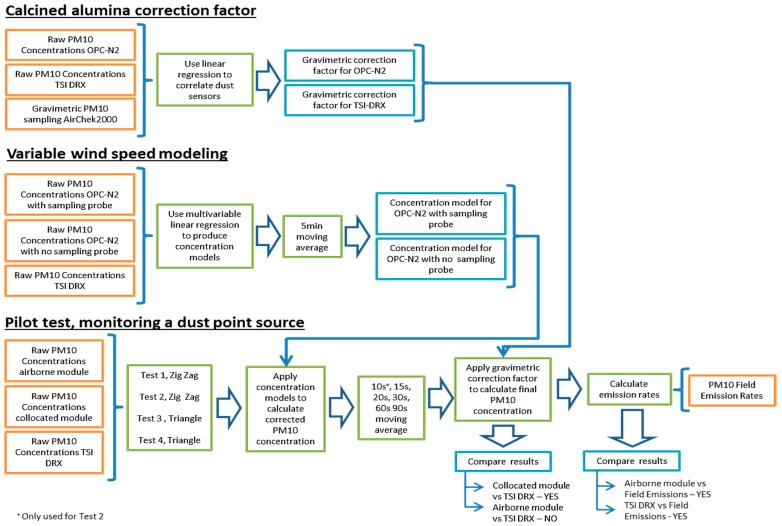
Flow diagram summarizing tests undertaken to validate readings collected with the dust monitoring sensors.

In Figure 8, the process to obtain the correction factors is presented as a flow diagram. This diagram also summarises the other two experiments conducted to validate the readings of the dust monitoring sensors. These experiments are described in the following sections.

### 2.4. Variable Wind Speed Modeling

**Figure 9:**
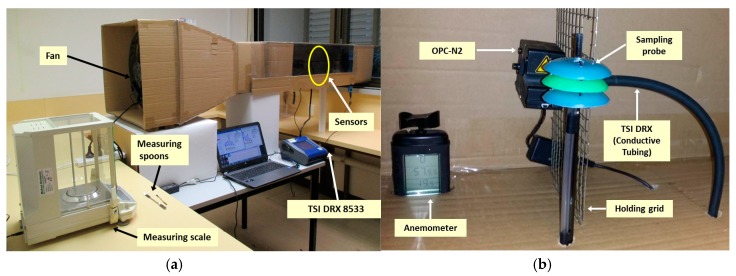
Wind tunnel and equipment used for the variable wind speed tests, (**a**) overall view of the wind tunnel and equipment used; and (**b**) test area set with the anemometer, probe, OPC-N2 and TSI DRX.

In addition to the previous test a variable air speed experiment was designed to study the response of the OPC-N2 with the probe attached using different wind speeds. For this experiment a wind tunnel was built to produce a laminar flow and have control of the air speed changes (Figure 9). The test area of the wind tunnel was 40.0 cm cross section, in which the probe (attached to the OPC-N2), a secondary OPC-N2 without a probe, and the TSI DRX (making use of the conductive tubing), were placed at the same height (15.0 cm from base of test area) for uniformity.

Four individual experiments were conducted to generate a variable wind speed model. Each experiment used a different wind speed: 1.11 m/s, 1.67 m/s, 3.06 m/s and 3.89 m/s. The air flow was produced with fans and measured with a SkyWatch ATMOS anemometer (Sudbury, UK). Dust particles were supplied using calcined alumina at an approximate rate of 0.015 g every 10 min. The concentration read by the OPC-N2 was logged and reported by the software interface provided by Alphasense Ltd. (Great Notley, Essex, UK).

Two equations were generated out of this experiment, one for the OPC-N2 connected to the probe and one equation for the OPC-N2 which was collocated with the TSI DRX during the flight tests. The resulting coefficients for the airborne module system equation and for the collocated module are shown in Equations (1) and (2) and Table 2:

C_UAV_ = (2.118 × C_R_) – (1.175 × R.H.) – (1.261 × T) + (1.822 × U) + 102.082 (1)

C_Coll_ = (0.197 × C_R_) – (0.623 × R.H.) – (0.641 × T) + (1.125 × U) + 57.292 (2)

The R^2^ for both equations using a multivariate linear regression was above 0.5. The airborne module had a coefficient of 0.55 and the collocated module sensor of 0.66. Coefficients with a *p*-value < 0.05 (95% confidence) is regarded as statistically significant. Coefficients within a 95% confidence level interval indicate they have a strong influence in the model outcome [19]. For Equations (1) and (2), all variables have a confidence level of approximately 100% (calculated as 1-*P* and expressed as a percentage). The model produced reliable results when used to correct raw data collected from the dust monitoring modules. However, both equations were calculated without the use of the feeding dust rate, due to resulting overestimated values, with errors up to 1000% when applying the model to field data collected during the flight tests. A possible explanation to the high error produced could be that the dust was supplied manually and this could produce less precise measurements than using an automated system. Due to this limitation, the models generated did not include the dust supply variable (W). Nevertheless, results of the calculations were the models were used produced estimates with low percentage errors.

### 2.5. Particle Size Distribution for Variable Wind Speed Experiment

Research has demonstrated that factors like wind speed, wind direction, air intake angle and shape of the nozzle, among others, affect the proportion of particles that are measured by monitoring devices [12]. Due to these considerations, dust particles should be measured under isokinetic and iso-axial conditions. In order to assess how the particle size distribution of the air sampled would be affected by the probe and different wind speeds, the data was normalised based on a percentage calculated with the average quantity of particles counted in determined size ranges.

**Figure 10:**
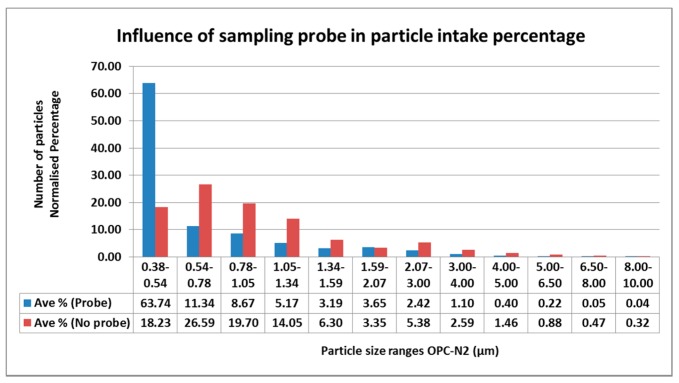
Influence of the sampling probe used with the OPC-N2 over particle counting readings per particle size range.

Figure 10 shows the normalized data for all wind speeds classified by particle size diameter (using the bins set by the OPC-N2). It is observed that the particles ranging from 0.38–0.54 µm are dominant when using the probe with a percentage difference of 45.5%. These are followed by particles ranging from 0.54–1.59 µm with an average percentage difference of 9.6%. This data indicates that up to a diameter of 2.0 µm, particles are expected to be in higher proportion that particles with larger diameter. Particles with a diameter from 1.59 µm to 10 µm presented a lower difference of 1.0%. Finally, the total difference estimated was of 7.6%. Through this analysis, it is possible to observe that particles up to approximately 2.0 µm are dominant when measuring with the sampling probe suggested in the methodology. However, results of the modeling estimates suggest that the effect of the probe can be corrected to obtain comparable results to field values.

**Figure 11:**
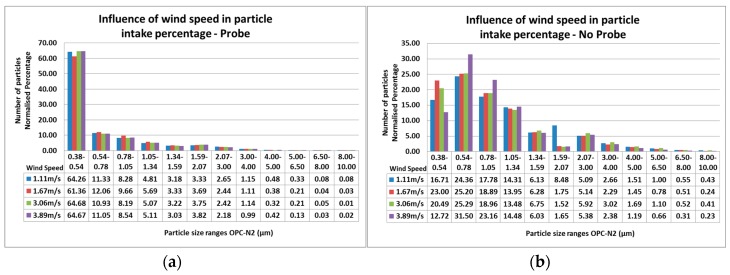
Influence of wind speed variation over particle counting readings per particle size range for (**a**) OPC-N2 with a sampling probe attached; and (**b**) OPC-N2 without a sampling probe.

Figure 11a,b shows the impact that variable wind speeds produce in the OPC-N2 by itself and with the sample probe attached. The distribution of particles counted by the sensor follows the same pattern that was observed in Figure 10. The average difference between wind speeds per bin is higher when not using the sampling probe, with difference of 10.3% in particles within 0.38–0.54 µm. The highest difference was also observed in the particle range from 0.38–0.54 µm when using the sampling probe. Impacts by the probe were expected, nevertheless as stated previously, by using the models it was possible to correct these effects in the sample intake and obtain results with low errors.

**Figure 12:**
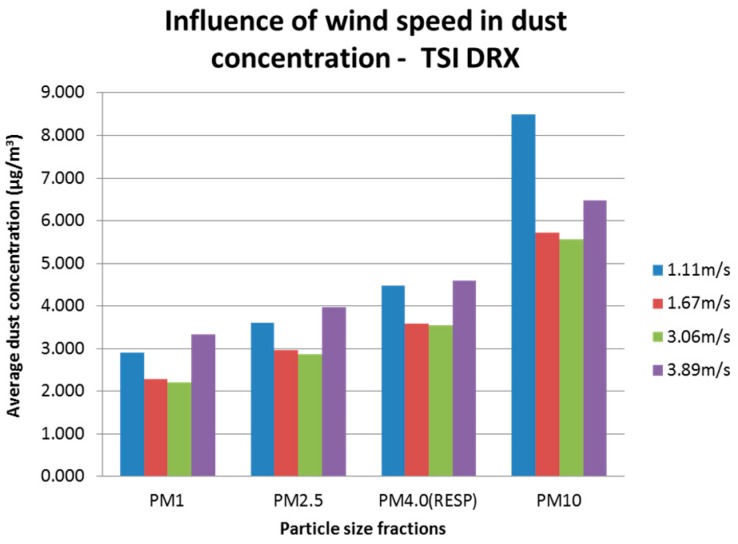
Influence of wind speed variation over dust concentration (raw) for different particle size fractions reported by the TSI DRX.

For the TSI DRX, the concentration for different particle size fractions was analysed. Particle fractions considered for the analysis were: PM_1.0_, PM_2.5_, PM_4.0_ (respirable according to ISO 12103-1, A1), and PM_10_. Figure 12 shows the averaged raw concentration per wind speed used. It is observed that the greatest difference between readings of a particle size with different wind speeds is produced by PM_1.0_ and PM_10_ fractions with values approximately 50.0% greater than the minimum average reading. Lower average concentrations were constant for wind speeds of 1.67 m/s and 3.89 m/s, and greater for wind speeds of 1.11 m/s and 3.89 m/s. These constant patterns observed in the different particle size fractions, produced a better outcome when generating the models.

## 3. Flight Tests, Monitoring a Dust Point Source

**Figure 13:**
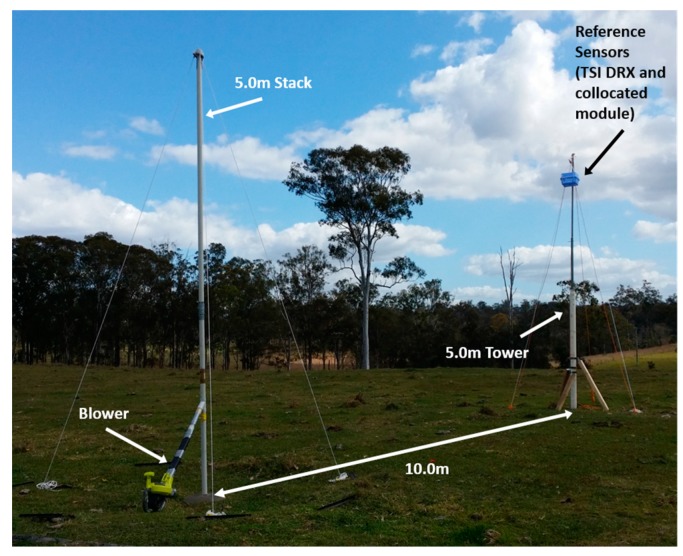
Experimental set up for the flight tests with the source stack and the monitoring tower.

An experiment was designed to collect experimental data similar to that obtained at open pit mine sites from activities such as hauling, stockpiling, blasting, etc. These activities are area sources or fugitive emissions, however to create an experimental site in which variables could be easily monitored and controlled, a stack emission was the most feasible option. The experiment consisted of using a 5.0 m stack (5.0 cm diameter), through which calcined alumina was supplied and expelled with the use of an electric leaf blower. Due to the dimensions of the stack, it was not possible to sample PM_10_ concentration readings from inside the stack isokinetically. However, this contributed in making the scenario similar to a real fugitive emission, measuring directly with the multi-rotor UAV from the plume generated. A 5.0 m tall “monitoring tower” was installed at a distance of 10.0 m downwind from the stack. At the top of the tower, a TSI DRX and a duplicate of the dust monitoring module were installed. This tower was used to compare dust concentration readings from stationary devices against airborne data (Figure 13).

A variable quantity of calcined alumina was blown out of the stack at approximately 30 s intervals. The total weight of powder expelled per interval was measured using an electronic balance (ELB600, Shimadzu, Kyoto, Japan). In-situ meteorological data was collected with an Oregon Scientific WMR200 weather station placed at the test site. Status and readings from the two modular sensors were followed via the radio link and controlled using SSH connection. In order to use the data produced by all the monitoring devices (dust monitoring modules, TSI DRX, and meteorological station) it was necessary to synchronise their datasets by programming the weather station and the TSI DRX. However, the dust monitoring modules had to be adjusted by internet clock link using WiFi and a portable hot-spot connection.

### 3.1. Procedure for Flight Tests 1 and 2

**Figure 14:**
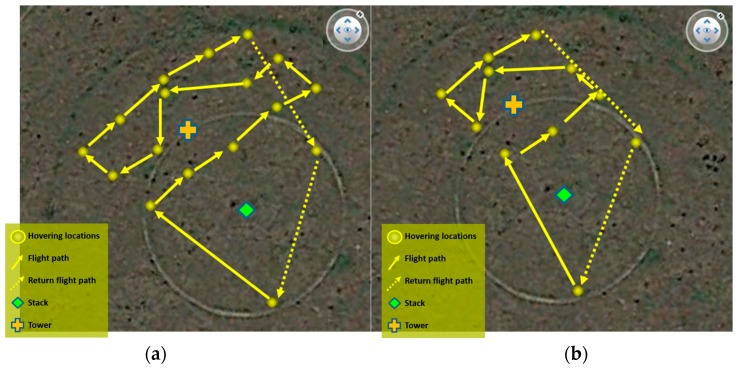
Characterizing zig-zag grids designed for (**a**) Test 1 and (**b**) Test 2.

In Alvarado et al. (2015), a circular flight pattern was used to scan the area and create a grid to characterize the dust plume. However, for this flight test a different grid was designed for this test due to the small size of the plume and the endurance of the UAV, which was of approximately 10 min. Two grids were designed for the first two tests. The grids were oriented northwest, downwind from the stack (source), and followed a zig-zag path around the monitoring tower (Figure 14).

**Figure 15:**
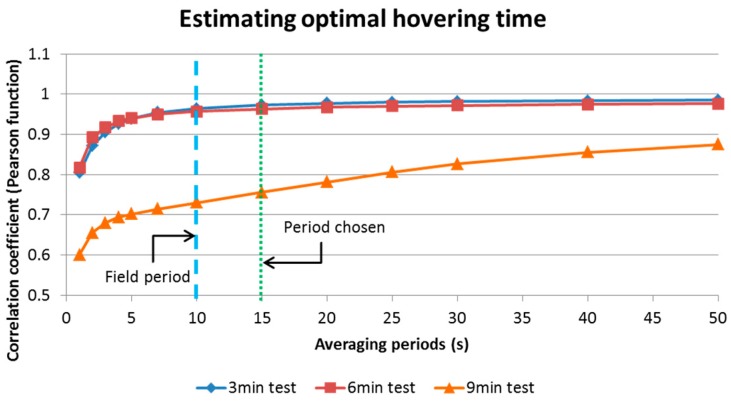
Calculated correlation coefficients (*r*) for different averaging periods of PM_10_ concentrations for three sets of tests.

For Test 1 the multi-rotor UAV was programmed to follow a predetermined path using the “Tower” mobile application from 3DR, hovering at waypoints for 15 s. The pattern was repeated at heights of 7.0 m and 9.0 m covering an area of 225.0 m^2^ approximately. The hovering periods were estimated on 15 s lapses according to the analysis of data produced in the laboratory during the calibration tests. This was estimated by averaging concentrations at different time periods and obtaining their correlation (linear association) using the Pearson product-moment correlation coefficient (*r*). Making a plot of the coefficients, it was possible to choose a time period that could reduce noise in the readings produced by the OPC-N2, and also allowed enough readings to be collected during the 10 min flights (Figure 15).

However, once in the field it was observed that the battery did not last long enough to cover the flight programmed. A second grid was designed for Test 2. For this test, the multi-rotor UAV hovered at the waypoints for 10 s, repeating the pattern at heights of 7.0 m, 9.0 m and 11.0 m. The grid covered an approximate area of 130.0 m^2^. For this experiment, the OPC-N2 software interface was not used to log the resulting concentration. A custom made program was written to record the data from all sensors that integrated the dust monitoring module. The OPC-N2 provided the particles counted for 16 predetermined bins, from which only 12 where considered due to the particle size targeted (PM_10_) (see Table 3).

To calculate the concentration for each reading produced by OPC-N2, the total bin volume of particles was estimated and multiplied by the particle density with a value of 1.65 × 10^12^ µg/m^3^ [11] to determine the total dust mass. The total mass was divided by the total volume of air sampled to calculate the final concentration. The total volume of air sampled was estimated with the sampling period and the sample flow rate (data provided by the sensor). Sampling period was set to 1 s as all data was averaged to match the sampling period of the TSI DRX.

### 3.2. Procedure for Flight Tests 3 and 4

Tests 3 and 4 followed a different flight strategy with the objective to only collect data flying next to the monitoring tower to determine the best way to validate readings from the dust monitoring module. These tests consisted of hovering close to the sides and behind the collocated devices in a triangular pattern. The flight was made in hover (loiter) mode and controlled manually. In total 4 flights were undertaken, two with a zig-zag pattern and two with a triangle pattern, each one of them with a total duration of 10 min approximately. Table 4 summarizes the characteristics of the four tests conducted for this section.

## 4. Flight Test Results

### 4.1. Results and Analysis of Data Collected with the Collocated Module

Linear multivariate regression was used to observe the relation between variables and obtain the correlation between the TSI DRX and the two dust monitoring modules. The variables included in the analysis were: air speed (m/s), relative humidity (%), temperature (C), raw concentration (µg/m^3^), and emission rate (µg/s). When the raw data was used as an input no correlation was observed. However, a better correlation was found by using the moving average of the concentrations. This was done in a similar way to how the OPC-N2 reports readings in its standalone interface (provided by Alphasense Ltd.) using a 5 min moving average.

For all tests, moving averages of 15 s, 20 s and 30 s were used to determine the best fit for the linear multivariate regression (Section 2.3). This resulted in having 20 s–30 s periods with the highest correlations with R^2^ of up to 0.8 for the collocated module. Table 5 and Table 6 presents a summary of the different approaches used to correlate data collected with the TSI DRX and the collocated module in the four tests analysed.

**Figure 16:**
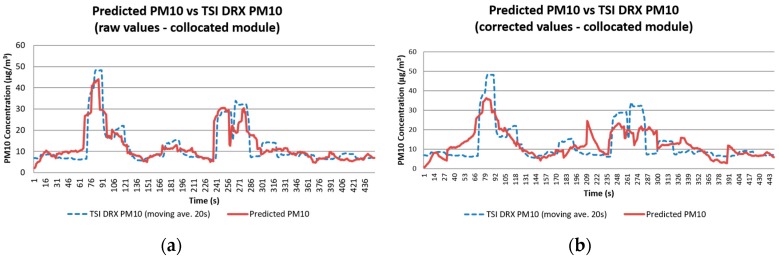
Comparison between PM_10_ concentration readings obtained with the TSI DRX and the predicted values calculated using the models generated for Test 2 with (**a**) raw values; and (**b**) corrected data.

Equations (1) and (2) were used to correct the raw concentrations. It is observed that the R^2^ values for correlations made with corrected concentrations, when compared against R^2^ values from regressions made with raw concentrations, were very close. This indicated that a model can be generated without making the correction. However, the use of corrected values was necessary when calculating emission rates. Figure 16 presents the values predicted using the model generated for Test 2, compared with the readings from the TSI DRX. An R^2^ of 0.74 (moving average of 20 s) was obtained with linear regression. By using a moving average of 30 s the R^2^ can be improved to 0.8, reducing greatly the noise observed in the corrected values. Such R^2^ indicates that the sensor module design can obtain reliable PM_10_ readings comparable to a TSI DRX in the field when placed at the monitoring tower.

From Table 4 and Table 5 and comparing R^2^ from Tests 1 and 2 against Tests 3 and 4, it was observed that Tests 1 and 2 have higher values. This could indicate that the multi-rotor UAV was flown closer than necessary to the tower, affecting the natural flow of the plume across the reference devices.

### 4.2. Comparison of Average Concentrations, Airborne Module Results Tests 2

Table 7 presents the correlation between the data collected with the airborne module for Test 2 and the TSI DRX. A linear multivariate regression was also performed for this dataset. The data is presented as an example to show that models created for the airborne module whilst traveling to different waypoints during the test do not have a high correlation with data collected with the TSI DRX. A statistical analysis was conducted to investigate if the model could be improved by including other variables that affect the dispersion of dust, such as wind direction and UTM coordinates of the UAV. However, when the model was generated and used, the resulting predicted values were not correlated and produced a R^2^ of 0.1–0.2.

**Figure 17:**
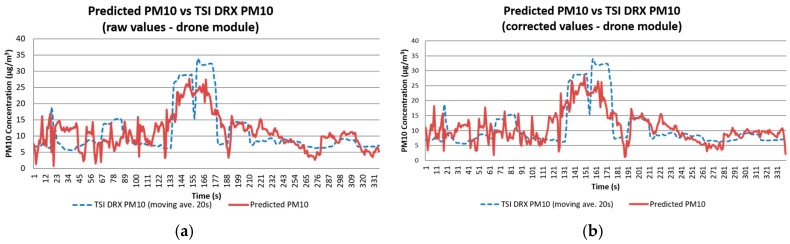
Comparison between PM_10_ concentration readings obtained with the TSI DRX and the predicted values calculated for Test 2 using the models generated with (**a**) raw values; and (**b**) corrected data.

Figure 17 presents the results of the concentrations calculated using the corrected concentration and raw field values. The comparison between TSI DRX values and the predicted TSI DRX values presents noise, and their R^2^ indicates that 40%–60% of the data is represented by the model created. For Figure 17a with corrected values, errors were identified due to their negative value, and removed. The regression was recalculated obtaining an R^2^ of 0.53. This value is closer to the coefficient obtained with the regression generated with the raw concentration values.

The confidence level for all the variables used in the correlation of the raw data, was above 95%, indicating their high significance in the model to calculate dust emission concentrations. It was observed that for the emission rate field values, even though they had a value greater than 95%, by excluding this value from the model equation the resulting corrected values (used to calculate concentrations or emission rates) had a better fit. As mentioned before, this variation could be due to manual recording of the feeding rate.

### 4.3. Comparison of Average Emission Rates, Airborne Module Results Tests 1 and 2

**Figure 18:**
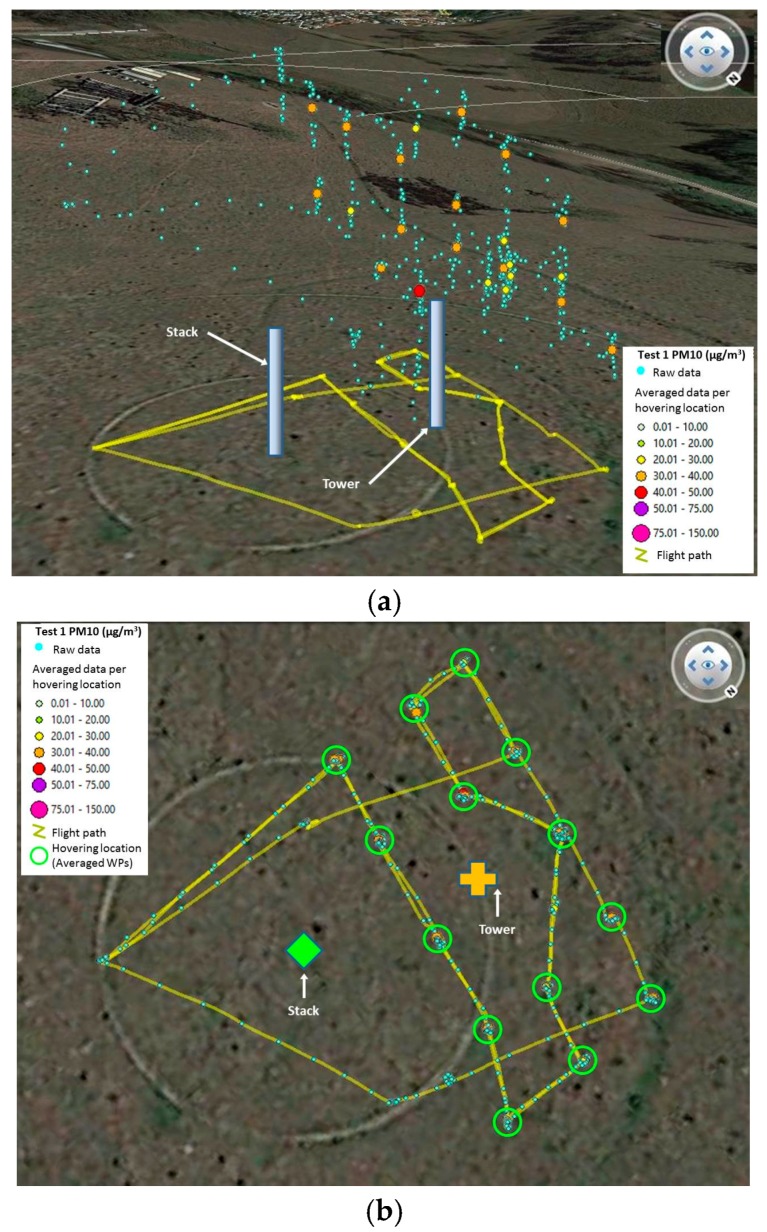
Field data collected for Test 1 with the airborne module before and after processing, (**a**) 3D view; and (**b**) top view with averaged locations indicated (repeated per height programmed).

**Figure 19:**
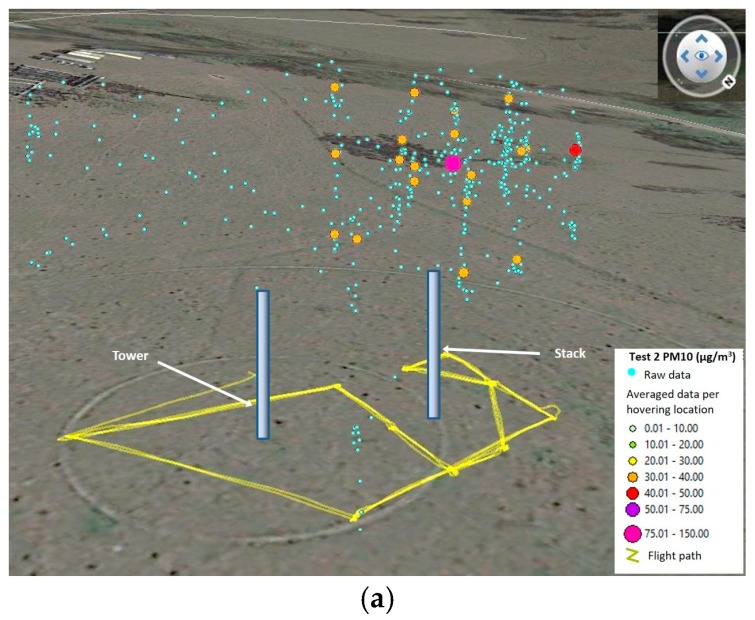

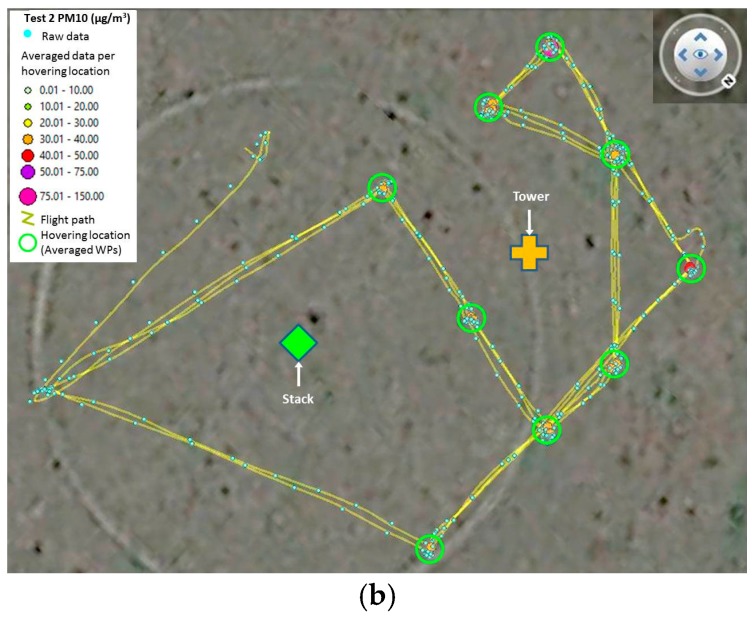
Field data collected for Test 2 with the airborne module before and after processing, (**a**) 3D view; and (**b**) top view with averaged locations indicated (repeated per height programmed).

Analysis of the zig zag dataset collected with the airborne module required isolating groups of waypoints corresponding to the 10 s and 15 s hovering periods programmed, and then averaging them by 10 s and 15 s, respectively. Once again, the moving average of the TSI DRX values together with the corrected values of the airborne module were calculated for periods of 10 s (only Test 2), 15 s, 20 s and 30 s. The percentage error between the reference device and the airborne module was calculated, to determine if the corrected concentration would be representative for the analysis. A total of 23 averaged hovering positions were used for Test 1 (Figure 18). Two thirds of 24 hovering positions collected were used from Test 2 (Figure 19). The hovering positions not considered for analysis were left out due to errors in the logging of data with the airborne module.

As discussed in Section 4.2, concentrations of the airborne module are not considered comparable to the TSI DRX in Tests 1 and 2. Therefore, to validate the field values obtained, the average emission rate for the TSI DRX and the airborne module were calculated. Table 8 presents the results of the emission rates obtained with the readings of the TSI DRX and the corrected values of the airborne module. The emission rate was calculated using the general Gaussian equation for sources at ground level [20]:

Q = C × 2π × σy × σz × U,
(3)

With Q being the emission rate in µg/s, C is the concentration in µg/m^3^, σy and σz the dispersion factors for a puff emission, and U the wind speed in m/s. A Gaussian model was used for this investigation due to its simplicity and extensive use for different modeling scenarios [21,22,23]. Background levels were estimated by reviewing the data from all flight tests and checking the minimum concentrations registered, which ranged from 0.0 µm/m^3^ to 5.6 µm/m^3^.

The values collected with the TSI DRX in Test 1 presented errors ranging from 8.9% to 192.9% when compared against an estimated average field emission rate of 20,449.15 µg/s.

The lower value corresponded to a moving average of 20 s. The percentage errors produced by comparing the airborne module against the field emission rate have smaller differences ranging from 8.9% to 9.8%. The lowest percentage error corresponds to the emission rate calculated with a moving average of 90s. A high variability in the emission rates was observed between values calculated with the TSI DRX and airborne module.

Calculations of the emission rate for Test 1 were performed considering an atmospheric stability class D. The average wind speed of 3.0 m/s observed in this Test and this should correspond to a stability class B. However, wind speed values were measured at a height of 3.0 m (instead of using the standard height of 10.0 m), and friction with the ground would slow the wind down resulting in a different atmospheric stability. Considering this situation, a stability class D, better described the atmospheric conditions. Emission rates calculated with stability class B parameters produced errors ranging from 150% to 1333%, hence being considered not applicable for the analysis and interpretation of data.

Emission rates were calculated using stability class D parameters, having an average wind speed of 5.3 m/s for Test 2. Error values varied from 144.6% to 181.7% between emission rates calculated with the concentrations from the TSI DRX and the airborne module (Table 8). However, when comparing the emission rates against the average field emission rate of 15,383.67 µg/m^3^, the airborne module had percentage errors ranging from 18.5% to 28.4%. Even though these values overestimated the field value, they are closer than the emissions calculated using the values obtained by the TSI DRX located in the tower, ranging from 47.8% to 56.1%.

The raw values of the airborne module, for Tests 1 and 2, presented higher errors in their emission rates. This indicated that the correction made to the readings with the model developed in the laboratory, produced a better fit, having a higher correlation between the real value and the value obtained with the airborne module.

### 4.4. Comparison of Emission Rates, Airborne Module Results for Tests 3 and 4

**Figure 20:**
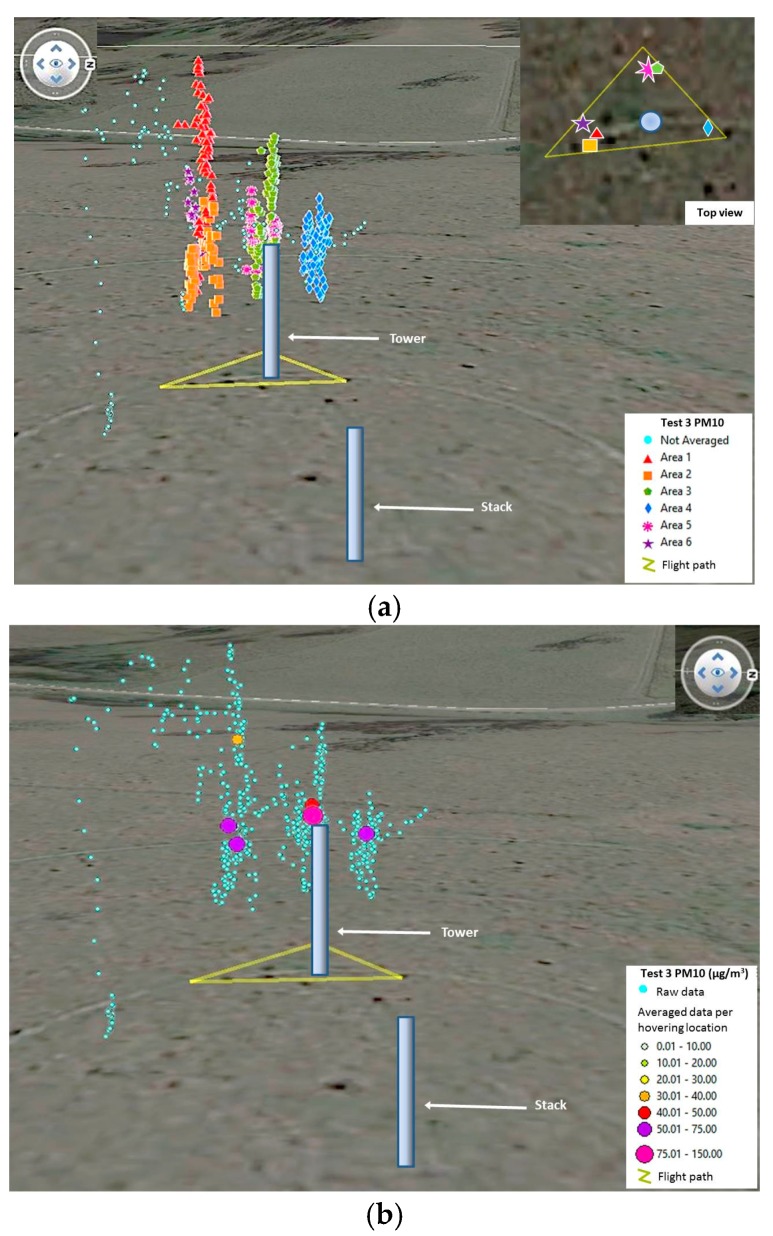
3D visualization for Test 3, showing (**a**) the waypoints grouped per high density areas; and (**b**) the raw data and waypoints used once averaged per area.

**Figure 21:**
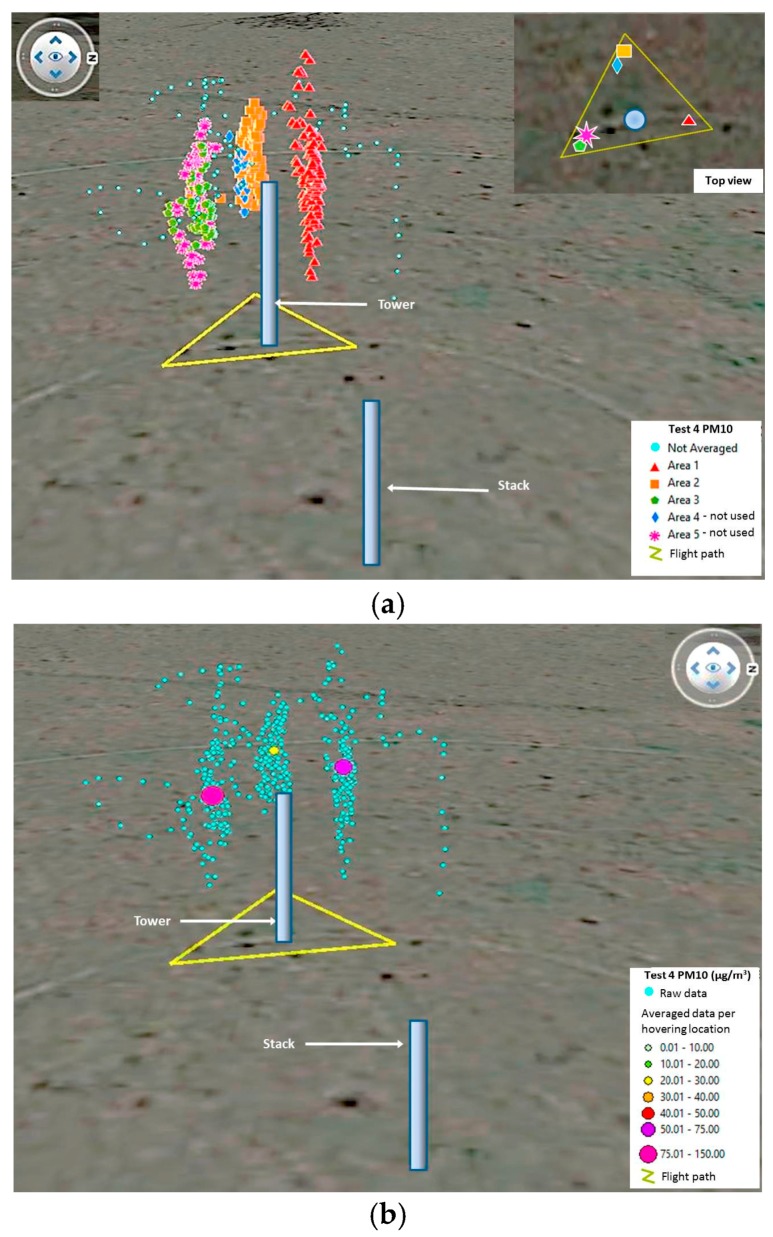
3D visualization for Test 4, showing (**a**) the waypoints grouped per high density areas; and (**b**) the raw data and way points used once averaged per area.

The triangle flight pattern tests required different approach for their analysis. The main objective was to collect as much data close to the TSI DRX and the collocated module by flying to the sides and behind the monitoring devices. Once the data was collected, it was grouped into high density areas. This resulted in six areas for Test 3 and five areas for Test 4 (Figure 20 and Figure 21). The moving average of all values was calculated considering intervals of 15 s, 20 s, 30 s, 60 s and 90 s. Then, each area was isolated and averaged per hovering period. This was done to determine the corrections with least error against the TSI DRX data. After observing the data in Test 4, it was considered necessary to eliminate an area located west of the devices and another located south of them, due to a high amount of blank readings (Figure 21). The other two areas located west and south of the devices presented very few blank logging records. All other areas selected for Tests 3 and 4 did not present drop-outs of sensor packets. The number of waypoints averaged per area defined in both tests ranged from 25 to 175, each waypoint representing a reading taken in one second.

Table 9 shows the resulting concentrations and emission rates obtained with the TSI DRX and calculated with the airborne module, as well as the calculated emission rate and their corresponding percentage error. The average estimated field value against which the airborne module values are compared was of 22,589.09 µg/s for Test 3 and of 50,864.86 µg/s for Test 4. For both tests, atmospheric stability class D was used in the calculation of emission rates, with average wind speeds of 5.2 m/s for Test 3 and of 5.1 m/s for Test 4. From Table 9 can be determined, in a similar way to Tests 1 and 2, that the values that produce the highest errors in the data are the raw values, indicating that the use of the moving average and the correction equation improved the precision of the readings considerably.

Emission rates calculated with the airborne module obtained errors as low as 1.2% in Test 3, and had the highest error in Test 4 at 43.3%. Percentage errors observed in Test 4 are considerably higher than errors presented by Test 3. This could be due mainly to the amount of data available to calculate a final emission rate with Test 3 having a complete dataset, and Test 4 having many points not considered due to errors in the log files. However, even with less data available the airborne module had a minimum error of 40.8% which is similar to errors obtained in the other tests with the TSI DRX, indicating data can still be considered useful.

Average concentrations obtained with the TSI DRX and the airborne module for both tests, have similar errors for moving averages of 15 s to 30 s, with higher variability in the 60 s and 90 s periods. This could be due to the lower amount of information in Test 4. The same behavior was also observed in Tests 1 and 2. This can lead us to the conclusion that 15 s to 30 s periods can be chosen as the preferred time periods.

## 5. Discussion

Analysis of the four available datasets demonstrated that very good estimations could be calculated, with percentage errors as low as 1.1% when compared to field emission rates. The use of different grids, hovering periods, moving average periods and the collection of environmental parameters, as well as UAV telemetry data from several sources, produced a very rich dataset.

Analysis of the particle size distribution during the variable wind experiment demonstrated that particles with a diameter up to approximately 2.0 µm dominated the sample when using the sampling probe. An average difference of 7.6% was observed to affect PM_10_ readings when the sampling probe was used and similar behavior was observed when analyzing the effect of variable wind speed in the OPC-N2 with and without the sampling probe. The particles with diameters ranging from 0.38–0.54 μm were also dominant. However, results from the models generated suggest that these impacts where corrected to obtain comparable results with field values.

Having observed that correlations were only possible if a moving average function analysis was used, different time periods were investigated to balance noise reduction, flight time and area covered. Moving averages of 15 s to 30 s produce consistent results and had some of the lowest percentage errors. On the other hand, it was observed that periods of 60 s to 90 s can produce noticeable variability due to the constant movement of the multi-rotor UAV which increased the difference between data collected as the sampling period increased.

It was expected that a moving average of 15 s or 10 s would produce a better correlation for Test 1 and Test 2 respectively, as these were the hovering sampling periods for which they were programmed. However, it was observed that only Test 2, with the airborne module, produced a reasonable correlation. This could be due to the influence of other environmental variables such as wind speed, wind direction, dust supply rate, etc. For both Tests 1 and 2, the raw values of the airborne module presented the higher errors in the calculation of emission rates. This indicated that the correction made to the readings with the model developed in the laboratory does increase the correlation between the real value and the value obtained with the airborne module. Lower percentage errors resulted when correcting the values calculated with the airborne module with Equations (1) and (2), due to an overestimation observed in all values once processed, when compared with the values of the TSI DRX.

The lowest errors produced when comparing calculated emission rates from the airborne module and the field values, were produced by Test 1 and Test 4. These results indicate that by hovering for short periods of time (10 s to 15 s) it is possible to produce estimations very close to the field values. Another advantage of hovering for short periods of time is that the battery can be used more efficiently, and more hovering locations can be covered within its flying endurance. Having the UAV hovering close to the devices in the tower can assist in concluding that the concentrations corrected with the model developed in the laboratory and the field values were comparable, with a low error between them.

It was possible to identify sources of error, such as: the synchronization of sensors and devices, calibration of sensors, calculation of correction factors and models, individual specifications of sensors (response time, precision, and resistance to exposure in the environment) through the analysis of all data. However, errors obtained for the tests analysed were low and it was important to consider that the methodology tested was a relative monitoring procedure; and not an absolute method such as a gravimetric test.

## 6. Conclusions

Four datasets were analysed for this investigation. With the current findings, it can be concluded that calculation of emission factors for specific activities in the industrial sector is possible making use of UAV technology. By using the proposed methodology, it is possible to generate emission factors per mine site and activity targeted.

The results of the four tests indicate that the airborne module can be used to obtain PM_10_ emission rates comparable to a monitoring system located in a tower with the major advantage that the airborne module can be transported to any location, at any time, weather conditions permitting.

Work will continue to include other variables in the modeling analysis. This includes micro and local meteorological data, more telemetry data, and a different modeling approach using artificial neural networks to include more variables. Artificial neural networks will increase the reliability of the model not only by linking a greater number of significant variables but also by providing feedback from new datasets generated.

## Figures and Tables

**Table 1:** Correction factors calculated with the analysis of the data from the dust chamber.

Device	TSI DRX		AirChek2000	
	Correction Factor	R^2^	Correction Factor	R^2^
OPC N2 (airborne module)	0.342	0.62	1.362	0.73
TSI DRX	NA	NA	1.312	0.92

NA = Not Applicable.

**Table 2:** Resulting coefficients for variables of equations for dust monitoring sensors with their *p*-value and confidence level.

Sensor	Variable	Coefficients	*p*-Value
Airborne module (C_UAV_)	Intercept	102.082	2.5 × 10^−223^
	Raw Conc. PM_10_ (C_R_, µg/m^3^)	2.118	~0.0
	Relative Humidity (R.H. %)	−1.175	4.2 × 10^−235^
	Temperature (T, °C)	−1.261	2.1 × 10^−231^
	Air speed (U, m/s)	1.822	2.8 × 10^−159^
	R^2^	0.55	NA
Collocated module sensor (C_Coll_)	Intercept	57.292	2.7 × 10^−84^
	Raw Conc. PM_10_ (C_R_, µg/m^3^)	0.197	~0.0
	Relative Humidity (R.H. %)	−0.623	5.6 × 10^−79^
	Temperature (T, °C)	−0.641	1.7 × 10^−72^
	Air speed (U, m/s)	1.125	2.0 × 10^−74^
	R^2^	0.66	NA

NA = Not Applicable.

**Table 3:** Bins considered to calculate PM_10_ concentrations and their boundaries according to particle diametre.

Bins	Bin Low Boundary, Particle Diametre (µm)	Bin High Boundary, Particle Diametre (µm)
0	0.38	0.54
1	0.54	0.78
2	0.78	1.05
3	1.05	1.34
4	1.34	1.59
5	1.59	2.07
6	2.07	3.00
7	3.00	4.00
8	4.00	5.00
9	5.00	6.50
10	6.50	8.00
11	8.00	10.00

**Table 4:** Characteristics of the four flight tests conducted.

Test	Pattern	Flight Mode	Hovering Periods (s)	Approximate Area Covered (m^2^)	Heights Flown (m)
1	Zig-Zag	Auto (programmed)	10	225.0	7, 9
2	Zig-Zag	Auto (programmed)	15	130.0	7, 9, 11
3	Triangle	Manual	Variable	10.6	5 m approx.
4	Triangle	Manual	Variable	10.2	5 m approx.

**Table 5:** Models generated from data collected with the TSI DRX and the collocated module using corrected concentrations and field data for Tests 1 and 2.

	Corrected Concentration Test 1			Raw Concentration Test 1			Corrected Concentration Test 2			Raw Concentration Test 2		
	Coefficients for Different Moving Averages											
**Variables**	15	20	30	15	20	30	15	20	30	15	20	30
Intercept	3527.905	3806.125	4339.276	2916.909	*1225.689*	−*32.446*	−734.98	−670.73	−413.48	−311.39	−279.21	−194.93
Air speed (m/s)	−28.921	−*15.235*	*8.638*	−32.403	−20.484	−11.931	−5.052	−3.76	−1.56	0.53	0.74	0.84
Rel. Humidity (%)	−53.060	−65.410	−84.957	−*17.782*	0.900	14.214	6.08	4.89	1.12	2.74	2.43	1.07
Temperature (°C)	−63.276	−55.941	−46.391	−78.939	−*46.455*	−21.134	15.65	15.79	13.55	7.37	6.60	5.58
Conc. PM_10_ (µg/m^3^)	25.568	25.720	23.828	5.347	6.200	6.584	4.85	3.69	1.40	1.77	1.88	1.88
Emission Rate (µg/s)	0.002	0.002	0.003	*1.0 × 10^−4^*	*−9.5 × 10^−5^*	*−2.0 × 10^−4^*	3.0 × 10^−4^	3.55 × 10^−5^	4.0 × 10^−4^	1.1 × 10^−4^	9.96 × 10^−5^	1.5 × 10^−4^
**R^2^**	0.61	0.64	0.61	0.67	0.81	0.89	0.46	0.54	0.59	0.63	0.74	0.80

Note: Numbers in *italics* indicate a confidence level lower than 95%.

**Table 6:** Models generated from data collected with the TSI DRX and the collocated module using corrected concentrations and field data for Tests 3 and 4.

	Corrected Concentration Test 3			Raw Concentration Test 3			Corrected Concentration Test 4			Raw Concentration Test 4			
	Coefficients for Different Moving Averages (s)											
**Variables**	15	20	30	15	20	30	15	20	30	15	20	30
Intercept	−3205.983	−2758.832	−2383.600	−2471.678	−2031.929	−1727.493	−2603.777	−2453.5	−1930.551	−2244.417	−2164.951	−1773.315
Air speed (m/s)	−70.601	−60.273	−48.545	−58.775	−49.292	−39.101	−9.773	2.415	−8.908	*3.379*	*3.002*	2.615
Rel. Humidity (%)	26.778	24.405	24.722	17.740	15.217	16.573	14.641	13.608	10.115	*9.471*	8.975	6.509
Temperature (°C)	83.074	67.556	52.224	80.139	64.912	48.573	69.263	65.052	50.422	71.857	69.719	58.798
Conc. PM_10_ (µg/m^3^)	16.947	16.412	13.282	80.139	3.383	2.839	14.059	14.349	14.679	2.813	2.916	3.058
Emission Rate (µg/s)	−0.003	−0.002	−0.001	3.396	−0.002	−0.002	3.0 × 10^−4^	3.0 × 10^−4^	8.66 × 10^−5^	4.0 × 10^−4^	3.0 × 10^−4^	2.0 × 10^−4^
**R^2^**	0.41	0.46	0.52	0.41	0.47	0.54	0.42	0.47	0.54	0.44	0.49	0.57

Note: Numbers in *italics* indicate a confidence level lower than 95%.

**Table 7:** Models generated with data collected with the TSI DRX and experimental modules using corrected concentrations and field data for Test 2.

	Corrected Concentration			Raw Concentration		
		Coefficients for Different Moving Average (s)				
**Variable**	15	20 *	30	15	20 *	30
**Airborne module**						
Intercept	296.29	319.96	214.72	182.79	221.04	182.19
Air speed (m/s)	*–0.46*	–0.77	–1.39	–1.91	–1.47	–0.67
Rel. Humidity (%)	–3.30	–3.58	–2.62	–2.35	–2.81	–2.44
Temperature (°C)	–3.66	–4.30	–3.01	–2.32	–3.06	–2.47
Conc. PM_10_ (µg/m^3^)	–1.4	–1.11	–0.04	–4.39	–3.04	–1.72
Dist. to source (m)	*	*	*	0.63	0.70	0.61
Emission Rate (µg/s)	0.00018	0.0002	0.0002	0.0002	0.0002	0.0002
**R^2^**	0.43	0.49 ^†^/0.54	0.45	0.45	0.52 ^†^/0.56	0.51

Note: Numbers in *italics* indicate a confidence level lower than 95%. * Distance value not used to obtain the best fit of the equation. ^†^ R^2^ value before errors were eliminated.

**Table 8:** Summary of concentrations and emission rates calculated for Tests 1 and 2 with their percentage error.

Measurement	Average Concentration (µg/m^3^)	Conc. Error (%), TSI DRX vs. Airborne Module	Emission Rate (µg/s)	Emission Error (%), Calculated Value vs. Field Value	Average Concentration (µg/m^3^)	Conc. Error (%), TSI DRX vs. Airborne Module	Emission Rate (µg/s)	Emission Error (%), Calculated Value vs. Field Value
	Test 1				Test 2			
**TSI DRX**								
Raw data	18.579	NA	10,646.214	47.9	14.339	NA	7213.353	53.1
10 s Mov. Ave.	NA *	NA	NA *	NA *	13.766	NA	6924.893	54.9
15 s Mov. Ave.	29.165	NA	16,712.084	18.27	13.874	NA	6979.260	54.6
20 s Mov. Ave.	32.500	NA	18,623.284	8.93	13.504	NA	6793.132	55.8
30 s Mov. Ave.	39.484	NA	22,625.078	10.64	13.414	NA	6747.759	56.1
60 s Mov. Ave.	93.055	NA	53,322.700	160.76	14.377	NA	7232.394	52.9
90 s Mov. Ave.	104.552	NA	59,911.108	192.98	15.952	NA	8024.461	47.8
**Airborne module**								
Raw data	3.201	82.7	1834.189	91.0	1.549	89.29	779.523	94.9
10 s Mov. Ave. Corr.	NA *	NA *	NA *	NA *	36.245	163.39	18,232.334	18.5
15 s Mov. Ave. Corr.	32.336	10.9	18,529.561	9.4	36.373	162.2	18,296.784	18.9
20 s Mov. Ave. Corr.	32.129	1.1	18,410.932	9.8	36.559	170.7	18,390.435	19.6
30 s Mov. Ave. Corr.	32.357	18.8	18,541.318	9.3	37.782	181.7	19,005.532	23.5
60 s Mov. Ave. Corr.	32.382	65.2	18,555.661	9.3	39.258	173.1	19,748.159	28.4
90 s Mov. Ave. Corr.	32.487	68.9	18,615.632	8.9	39.012	144.7	19,624.319	27.6

NA = Not Applicable. Mov. Ave. = Moving Average. Mov. Ave. Corr. = Moving Average of Corrected data from the airborne module. * A Moving Average of 10s was calculated only for Test 3 as the hovering period for each way point programmed was of 10 s, for Test 1 the hovering period was of 15 s.

**Table 9:** Summary of concentrations and emission rates calculated for the triangle test with their percentage error.

Measurement	Average Concentration (µg/m^3^)	Conc. error (%), TSI DRX vs. Airborne Module	Emission Rate (µg/s)	Emission error (%), Calculated Value vs. Field Value	Average Concentration (µg/m^3^)	Conc. Error (%), TSI DRX vs. Airborne Module	Emission Rate (µg/s)	Emission Error (%), Calculated Value vs. Field Value
	Test 3				Test 4			
**TSI DRX**								
Raw data	67.263	NA	25,328.05	12.1	76.610	NA	37,452.015	26.4
15s Mov. Ave.	57.832	NA	21,777.01	3.6	55.746	NA	27,252.411	46.4
20s Mov. Ave.	50.777	NA	19,120.22	15.4	54.135	NA	26,464.745	47.9
30s Mov. Ave.	43.630	NA	16,428.88	27.3	51.831	NA	25,338.413	50.2
60s Mov. Ave.	36.639	NA	13,796.32	38.9	51.197	NA	25,028.419	50.8
90s Mov. Ave.	34.733	NA	13,078.78	42.1	48.600	NA	23,758.769	53.3
**Airborne module**								
Raw data	9.2702	86.2	3490.715	84.5	8.464	88.95	4137.911	91.9
15s Mov. Ave. Corr.	61.399	06.2	23119.81	2.3	60.952	9.34	29,797.558	41.4
20s Mov. Ave. Corr.	59.276	16.7	22320.61	1.2	61.017	12.71	29,829.075	41.4
30s Mov. Ave. Corr.	55.987	28.3	21081.93	6.7	61.204	18.08	29,920.592	41.2
60s Mov. Ave. Corr.	55.455	51.4	20881.7	7.6	61.548	20.22	30,088.789	40.8
90s Mov. Ave. Corr.	54.424	56.7	20,493.45	9.3	59.052	21.51	28,868.344	43.3

NA = Not Applicable. Mov. Ave. = Moving Average. Mov. Ave. Corr. = Moving Average of Corrected data from the airborne module.
